# Increased γ-H2A.X Intensity in Response to Chronic Medium-Dose-Rate γ-Ray Irradiation

**DOI:** 10.1371/journal.pone.0045320

**Published:** 2012-09-18

**Authors:** Takashi Sugihara, Hayato Murano, Kimio Tanaka

**Affiliations:** 1 Department of Radiobiology, Institute for Environmental Sciences, Rokkasho, Kamikita, Aomori, Japan; 2 Tohoku Environmental Sciences Services Corporation, Rokkasho, Kamikita, Aomori, Japan; Shantou University Medical College, China

## Abstract

**Background:**

The molecular mechanisms of DNA repair following chronic medium-dose-rate (MDR) γ-ray-induced damage remain largely unknown.

**Methodology/Principal Findings:**

We used a cell function imager to quantitatively measure the fluorescence intensity of γ*-*H2A.X foci in MDR (0.015 Gy/h and 0.06 Gy/h) or high-dose-rate (HDR) (54 Gy/h) γ*-*ray irradiated embryonic fibroblasts derived from DNA-dependent protein kinase mutated mice (scid/scid mouse embryonic fibroblasts (scid/scid MEFs)). The obtained results are as follows: (1) Automatic measurement of the intensity of radiation-induced γ*-*H2A.X foci by the cell function imager provides more accurate results compared to manual counting of γ*-*H2A.X foci. (2) In high-dose-rate (HDR) irradiation, γ*-*H2A.X foci with high fluorescence intensity were observed at 1 h after irradiation in both scid/scid and wild-type MEFs. These foci were gradually reduced through de-phosphorylation at 24 h or 72 h after irradiation. Furthermore, the fluorescence intensity at 24 h increased to a significantly greater extent in scid/scid MEFs than in wild-type MEFs in the G_1_ phase, although no significant difference was observed in G_2_/M-phase MEFs, suggesting that DNA-PKcs might be associated with non-homologous-end-joining-dependent DNA repair in the G_1_ phase following HDR γ*-*ray irradiation. (3) The intensity of γ*-*H2A.X foci for continuous MDR (0.06 Gy/h and 0.015 Gy/h) irradiation increased significantly and in a dose-dependent fashion. Furthermore, unlike HDR-irradiated scid/scid MEFs, the intensity of γ*-*H2A.X foci in MDR-irradiated scid/scid MEFs showed no significant increase in the G_1_ phase at 24 h, indicating that DNA repair systems using proteins other than DNA-PKcs might induce cell functioning that are subjected to MDR γ*-*ray irradiation.

**Conclusions:**

Our results indicate that the mechanism of phosphorylation or de-phosphorylation of γ*-*H2A.X foci induced by chronic MDR γ*-*ray irradiation might be different from those induced by HDR γ*-*ray irradiation.

## Introduction

Radiation induces double-strand-breaks (DSBs) on DNA, and these DSBs are effectively repaired by several phosphoinositide 3 (PI3) kinases, such as ataxia telangiectasia mutated kinase (ATM) and DNA-dependent protein kinase (DNA-PKcs) [1]–[3]. Two main pathways for DSB repair, non-homologous-end-joining (NHEJ) and homologous recombination (HR), are activated in irradiated mammalian cells. DNA-PKcs is involved in NHEJ along with the DNA ligase IV/XRCC4 complex to repair the DSBs [4]–[5]. Several differences have been reported between the 2 repair pathways. For instance, DNA repair fidelity is lower in NHEJ than in HR [6]. Moreover, DSB repair by NHEJ is effective for all cell cycle phases [7], whereas that by HR occurs predominantly in the late S or G_2_ phases [8]. At the molecular level, both activated DNA-PKcs and ATM phosphorylate a unique C-terminal serine residue of histone-H2A.X (H2A.X) in response to DSB induced by high-dose-rate (HDR) γ*-*ray irradiation [9]–[11]. The number of phosphorylated H2A.X (γ*-*H2A.X) foci in cell nuclei serves as an efficient marker for scoring of radiation-induced DSBs [12]. By measuring the decay of ^125^I incorporated into cellular DNA, a direct correlation was observed between the number of H2A.X foci and the number of DSBs [13]. This result suggests that each focus may represent an individual DNA break; thus, measurement of γ*-*H2A.X foci formation may represent a suitable method for studying the effect of low-dose (LD) or low/medium-dose-rate (L/MDR) γ*-*ray irradiation. Evaluation of the biological effects of LD or L/MDR γ*-*ray irradiation is extremely important for risk assessment, particularly for nuclear power plant workers, medical radiologists and other exposed individuals. Few papers have addressed the relation between LD or L/MDR γ*-*ray irradiation and γ-H2A.X foci [14], [15]. Dividing human primary cells could repair DSBs induced by extremely LD γ*-*ray (0.0012 mGy) irradiation at a HDR, but non-dividing cells could not [14]. These results suggest that cell cycle phase is an important factor affecting the number of observed γ*-*H2A.X foci in LD irradiation. Moreover, γ*-*H2A.X foci formation was observed in human ATM-deficient cells exposed to MDR (0.018 Gy/h) γ*-*ray, although only few foci were observed in ATM-deficient cells exposed to HDR γ*-*ray irradiation [15]. This result supports the existence of an ATM-independent mechanism of γ*-*H2A.X phosphorylation in MDR- but not HDR-irradiated cells. Different cellular responses between MDR (0.024 Gy/h) and HDR γ*-*ray irradiation have also been observed in NHEJ-defective DT40 chicken or mammalian cells [16]. Although these cells were found to be highly sensitive to MDR, the mechanism of MDR γ*-*ray–induced γ*-*H2A.X foci formation in NHEJ-defective cells has not been studied. In our current study, we focused on analyzing the correlation between MDR γ*-*ray irradiation and γ*-*H2A.X foci formation in mouse embryonic fibroblasts (MEFs) derived from SCID mice carrying a point mutation in the gene encoding DNA-PKcs [17].

The average numbers of γ*-*H2A.X foci (foci/cell) in nuclei subject to HDR γ*-*ray irradiation can be scored relatively accurately, because they follow a Gaussian distribution. In contrast, we found that the number of γ*-*H2A.X foci induced by γ*-*ray at LD or L/MDR irradiation does not follow a Gaussian, but a binomial distribution, as most cells have no γ*-*H2A.X foci (data not shown). The number of cells without γ*-*H2A.X foci cause the binomial distribution. Although strong intensity foci induced by HDR γ*-*ray irradiation are easy to count, weak intensity foci, such as those induced by MDR γ*-*ray irradiation are relatively difficult to detect. Thus, in the latter case, once cannot safely conclude whether a cell contains uncountable foci or no foci at all. This indicates the need to define the intensity levels of cells without γ*-*H2A.X foci, to avoid inconsistent measurements of the number of MDR γ*-*ray-induced foci. In general, no unified estimation has been performed for the intensity value of uncountable γ*-*H2A.X foci among different laboratories. Thus, manual image counting of γ*-*H2A.X foci in LD or MDR γ*-*ray-irradiated cells can be problematic. Most LD or MDR γ*-*ray-irradiated cells have no γ*-*H2A.X foci, and manual-image-counting of those cells using different thresholds of fluorescent intensity by individual researchers gives different results. Thus, the results of LD or MDR experiments differ among different laboratories, making it necessary to accurately define the intensity of MDR γ*-*ray irradiation-induced γ*-*H2A.X foci in a non-arbitrary manner. Previously published papers measuring γ*-*H2A.X foci induced by LD or MDR irradiation have used averaged numbers of γ*-*H2A.X foci (foci/cell) and calculation of their statistical significance using a parametric *t-test* without consideration of the number of cells with no γ*-*H2A.X foci [14], [15], [18].

In the present study, we used a cell function imager (IN Cell Analyzer 1000) as a novel tool for automatic counting of γ*-*H2A.X foci intensity in over a thousand cells in a short time, and aimed to quantitatively analyze γ*-*H2A.X foci intensity in nuclei without human arbitrariness. We propose that measurement of γ*-*H2A.X fluorescence intensity using a cell function imager can replace manual image counting of γ*-*H2A.X foci. Finally, our study addresses the correlation between MDR γ*-*ray radiation dose and γ*-*H2A.X foci intensity.

## Results

### Dose-dependent Increase in the Intensity of γ-H2A.X Foci Induced by HDR γ-ray Irradiation

**Figure 1:**
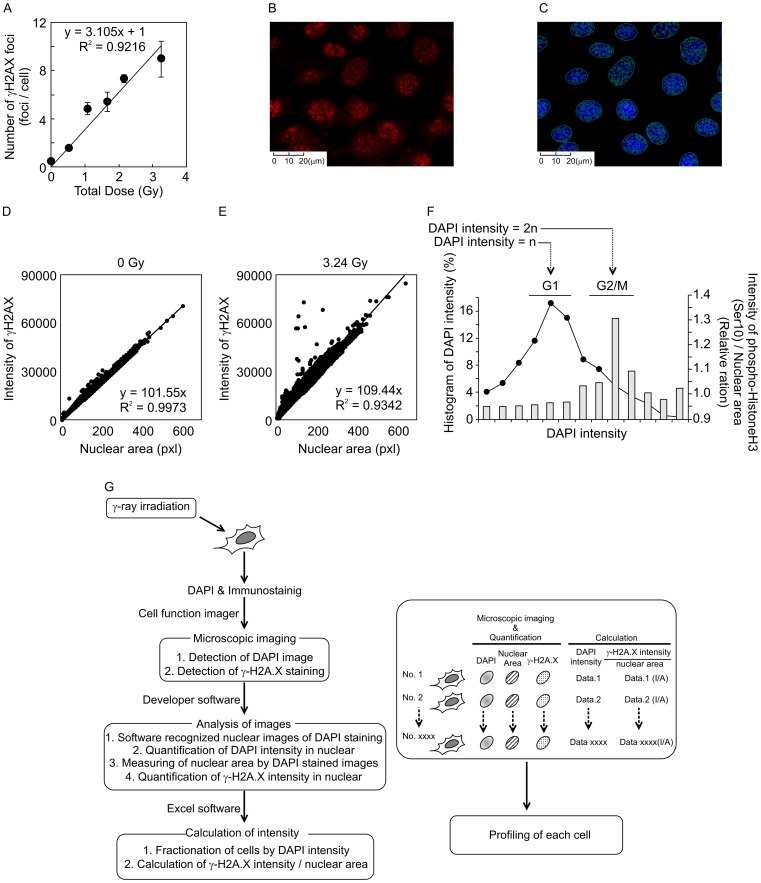
Detection of γ-H2A.X intensity induced by γ-ray irradiation. A. Number of γ*-*H2A.X foci (foci/cell) in wild-type (C.B.17/DNA-PKcs+/+Jcl) MEFs at 1 h after HDR γ*-*ray irradiation (0, 0.54, 1.08, 1.67, 2.16, and 3.24 Gy) was determined by manual image counting. Numbers of γ-H2A.X foci were counted in 100 cells at each point. These experiments were obtained by triplicated experiments. Average number of γ*-*H2A.X foci per cell in wild-type MEFs increased in a dose-dependent manner. B. Image of γ*-*H2A.X foci stained by anti-γ*-*H2A.X antibody and Alexa 647-conjugated anti-mouse-Ig G. C. Image of nucleus stained by 4′,6-diamino-2-phenylindole (DAPI). This image was automatically analyzed by a cell function imager (IN Cell Analyzer 1000). Circles traced in blue by the cell function imager indicate nuclei, and circles traced in green indicate γ*-*H2A.X foci. D. Relation between total fluorescence intensity of γ*-*H2A.X foci in nuclei and nuclear area, in non-irradiated cells. Nuclei are surrounded by a green line, as shown in Fig. 1B. Each point in this figure represents one detected cell. This result indicates that the intensity of γ*-*H2A.X foci per nuclear area (I/A) is constant in non-irradiated cells. E. Relation between total intensity of γ*-*H2A.X foci in nuclei and nuclear area, in cells irradiated by HDR at 3.24 G.y. F. Percentage of cell cycle fractionation in wild-type MEFs, with G_1_ and G_2_/M phases indicated by dot plots. Solid bars indicate intensity of phosphorylated histone H3 at Ser10. The relative ratios were calculated by stained intensity of phosphorylated histone H3 at Ser10 (median intensity of each cell fractions/average of total intensity of all cells). “n” indicates peak of DAPI intensity in G_1_ phase. “2n” indicates predicted peak in G_2_/M phase. G. A scheme of methods by cell function imager is summarized.

**Figure 2:**
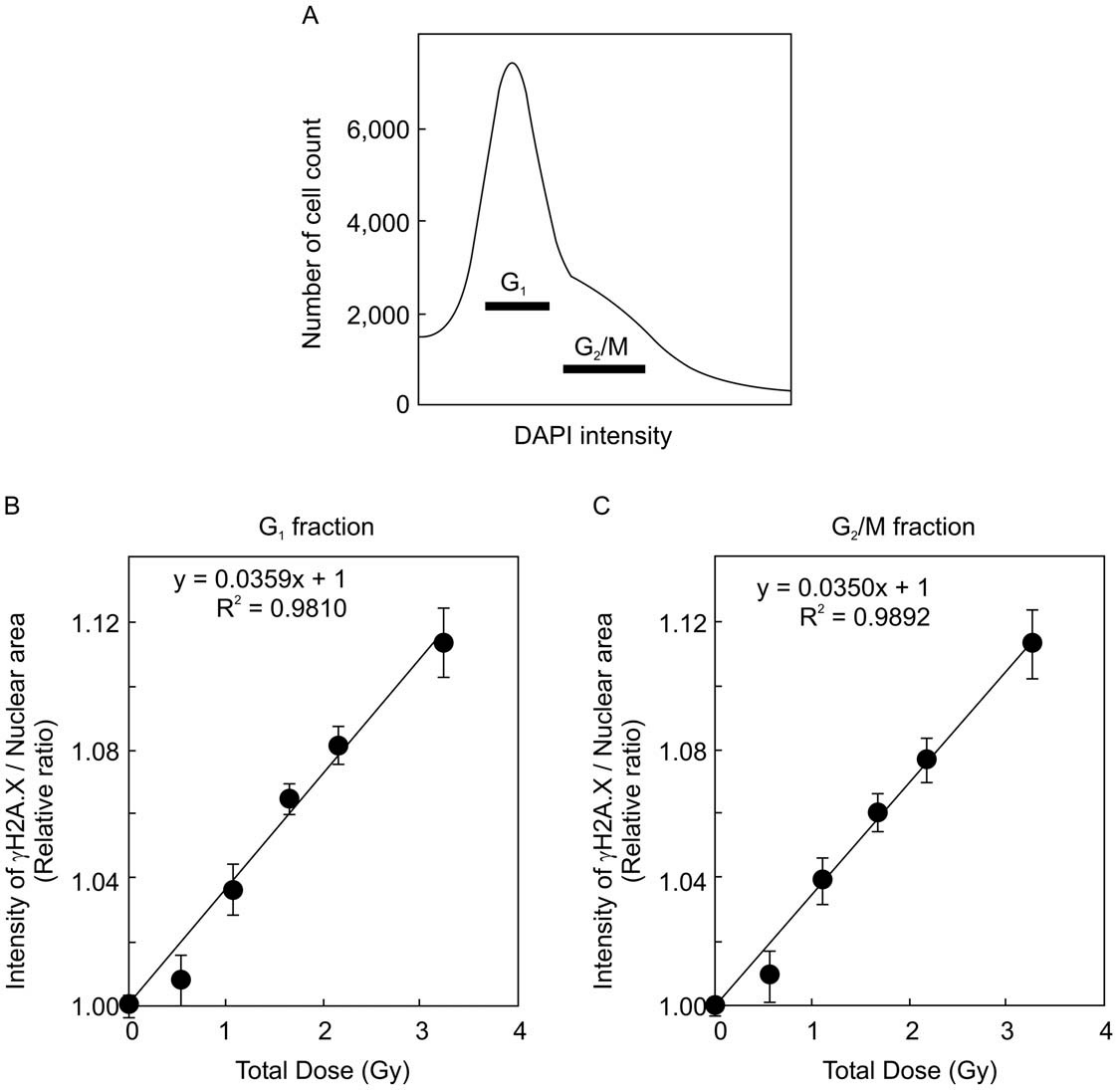
Increased intensity of γ-H2A.X foci induced by HDR γ-ray irradiation in wild-type MEFs. A. Histogram of cell cycle fractionation in wild-type MEFs, with G_1_ and G_2_/M phases indicated by black bars. B. Relation between averaged I/A (fluorescence intensity of γ*-*H2A.X foci per nuclear area) and total doses (0, 0.54, 1.08, 1.67, 2.16, and 3.24 Gy) by HDR irradiation in the G_1_-phase fraction of wild-type MEFs. Each point in the figure indicates a relative ratio (IR/non-IR: averaged I/A in the irradiated condition/averaged I/A in the non-irradiated condition), which is the representative value for each irradiation condition. This result indicates that the averaged I/A is increased dose-dependently by HDR irradiation. C. Relation between averaged I/A (γ*-*H2A.X foci per nuclear area) and total doses (0, 0.54, 1.08, 1.67, 2.16, and 3.24 Gy) by HDR irradiations in the G2/M-phase fraction of wild-type MEFs. Each averaged I/A was calculated from 10 replicate experiments (2 independent experiments with 5 points taken in each experiment) for detecting relative ratios.

**Figure 3:**
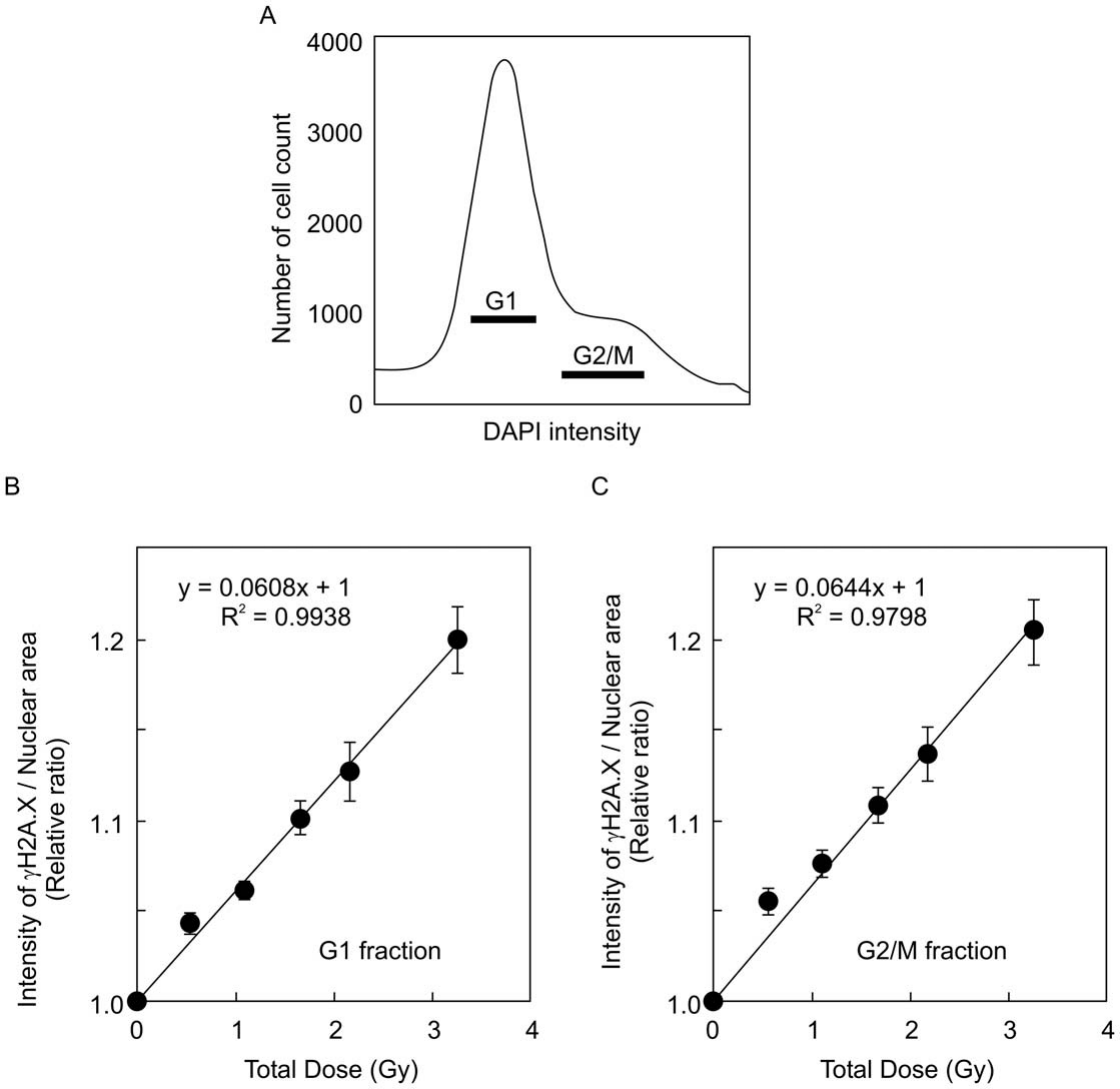
Increased intensity of γ-H2A.X foci induced by HDR γ-ray irradiation in scid/scid MEFs. A. Histogram of cell cycle fractionation of scid/scid MEFs, where the G_1_ and G_2_/M phases are indicated by black bars. B. Relation between averaged I/A (γ*-*H2A.X foci per nuclear area) and total doses (0, 0.54, 1.08, 1.67, 2.16, and 3.24 Gy) by HDR irradiation, in the G_1_-phase fraction from scid/scid MEFs. C. Relation between averaged I/A (γ*-*H2A.X foci per nuclear area) and total doses (0, 0.54, 1.08, 1.67, 2.16, and 3.24 Gy) by HDR irradiation in the G2/M phase fraction from scid/scid MEFs. Each averaged I/A was calculated from 10 replicate experiments (2 independent experiments with 5 points taken in each experiment) for detecting relative ratios.

The number of γ*-*H2A.X foci (foci/cell) in wild-type (FOX CHASE SCID C.B-17/lcr-^+/+^Jcl) MEFs at 1 h after HDR γ*-*ray irradiation was counted manually (Fig. 1A). The number of γ*-*H2A.X foci in a cell in the wild-type MEFs were increased in a dose-dependent manner, and their values at all doses showed a linear relationship (R^2^ values = 0.9216) by the least-squares method (Fig. 1A). Total fluorescence intensity in nuclei stained with the γ*-*H2A.X antibody was automatically measured by the cell function imager (IN Cell Analyzer 1000), and nuclear area and nuclear fluorescent intensity in each cell were determined from an image of DAPI staining at the same time (Fig. 1B, 1C). The values of nuclear areas and intensity of γ*-*H2A.X foci were interrelated in the same nucleus. Tens of thousands of wild-type MEFs were analyzed by cell function imager, and image intensity at all radiation doses (0, 0.54, 1.08, 1.67, 2.16, and 3.24 Gy) was measured by the Developer software. We examined whether background intensity levels of γ*-*H2A.X foci in nuclei increased analogously with nuclear area, as quantitated by DAPI staining. Our experiments revealed a linear relationship (R^2^ values = 0.9973) between γ*-*H2A.X fluorescence staining intensity and DAPI nuclear staining in the non-irradiated condition (0 Gy), as assessed by the least-squares method (Fig. 1D). This result indicates that the intensity of γ*-*H2A.X foci per nuclear area (I/A) depends on nuclear size in the non-irradiated MEFs. The calculated value for the slope using the least-squares method was 101.53 at 0 Gy, which was set as the baseline for background levels of γ*-*H2A.X foci (Fig. 1D). On the other hand, more variable values of I/A were observed in irradiated MEFs at 3.24 Gy, for which the calculated slope value by the least-squares method was 109.44, higher than that in non-irradiated cells (Fig. 1E). Average I/A value was higher for MEFs irradiated at 3.24 Gy than non-irradiated cells. We used the IR/non-IR relative ratio, obtained by dividing the average I/A in the irradiated state to the average I/A in the non-irradiated state [IR/non-IR =  (averaged I/A in irradiated condition)/(averaged I/A in non-irradiated condition)], to address the effect of radiation dose on foci formation. Using the *t-test,* we found a statistically significant difference between the relative ratio at 3.24 Gy (IR/non-IR = 1.24) and that of non-irradiated cells (non-IR/non-IR = 1.00). This result indicates that the relative ratio (IR/non-IR) can be used as a suitable parameter to quantitate radiation effects in the present experiments. A cell cycle histogram was drawn based on nuclear DNA content, as assessed by DAPI staining. We could distinguish between G_1_ and G_2_/M phases by DAPI intensity. To verify whether the G_2_/M fractions divided by DAPI staining is accurate or no, the G_2_/M phases were confirmed by relative ratios of phosphorylated histone H3 at Ser10 (a representative intensity in each fraction of histogram/average intensity of all cells), which was known as mitosis marker (Fig. 1F). These results indicated that G_2_/M phases by DAPI intensity is merged with phosphorylated histone H3 at Ser10. Thus, G_1_ and G_2_/M phases by DAPI intensity were suitable for cell cycle analysis. However, we could not detect sharp peak of intensity by DAPI. Therefore, our technique indicates only differences between intensities of γ-H2A.X fluorescence around G_1_-phase cells fraction, and those around G_2_/M-phase fraction. Further, we could not even distinguish G_1_-phase from G_0_-phase by this method. A scheme of these methods is summarized in Fig. 1G. A cell cycle histogram was drawn based on nuclear DNA content, as assessed by DAPI staining, and MEFs were fractionated into the G_1_ and G_2_/M phases, as indicated in Fig. 2A. Relative ratios (IR/non-IR) at each total dose were plotted for each cell cycle phase. The relation between relative ratio and total radiation dose was found to be linear using the least-squares method both in the G_1_ and G_2_/M phase (R^2^ values = 0.9810, R^2^ values = 0.9892, respectively, Fig. 2B, 2C). This result indicates that cell cycle phase has no effect on the relative ratio. We also measured the relative ratios in scid/scid MEFs at different high radiation doses (0, 0.54, 1.08, 1.67, 2.16, and 3.24 Gy). scid/scid MEFs were fractionated into the G_1_ and G_2_/M phase (Fig. 3A), and the relation between relative ratio and total radiation dose was found to be linear at both the G_1_ and G_2_/M phase (R^2^ = 0.9938 and R^2^ = 0.9798, respectively, Fig. 3B and Fig. 3C). These results indicate that relative ratio and radiation dose by HDR irradiation in MEFs show a linear correlation even in the absence of DNA-PKcs activity. Furthermore, relative ratios (IR/non-IR) derived from I/A both in the G_1_ and G_2_/M phases are suitable parameters that can be used to evaluate radiation effects.

### Time Course-dependent Change in γ-H2A.X Foci after HDR (54 Gy/h) γ-ray Irradiation

**Figure 4:**
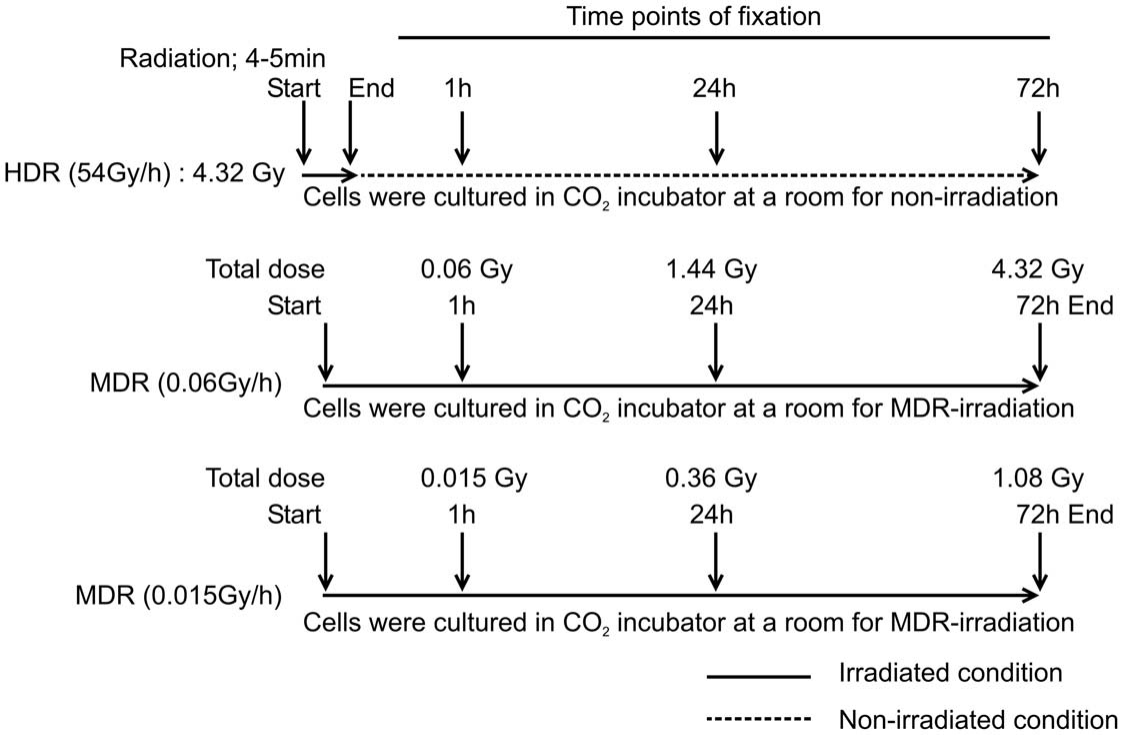
Schemes of γ-ray irradiations for detecting time-course-dependent change in the intensity of γ-H2A.X foci. HDR: 54 Gy/h (Total dose is 4.32 Gy), MDR: 0.06 Gy/h (Total doses are 0.06–4.32 Gy), MDR: 0.015 Gy/h (Total doses are 0.015–1.08 Gy).

**Figure 5:**
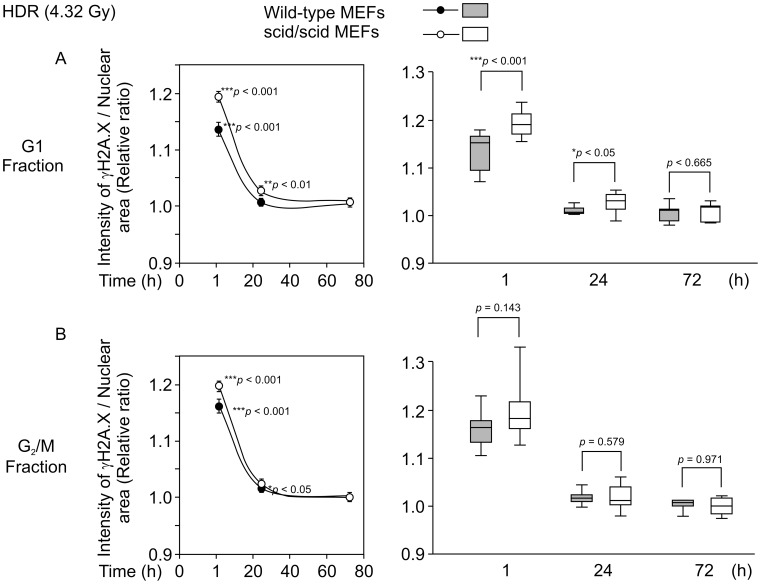
Time-course-dependent changes in the intensity of γ-H2A.X foci in response to HDR (54 Gy/h) γ-ray irradiation. A. The left side shows the relative ratios (IR/non-IR), at 1, 24, and 72 h after HDR (0.9 Gy/min, and total dose was 4.32 Gy) γ*-*ray irradiations in both wild-type and scid/scid MEFs from the G_1_-phase fraction. The right side shows the same data using a box plot, which depicts groups of numerical data through their five-number summaries, i.e., smallest observation, lower quartile, median, upper quartile and largest observation. B. The left side shows relative ratios (IR/non-IR) at 1, 24, and 72 h after HDR (0.9 Gy/min and total dose was 4.32 Gy) γ*-*ray irradiation in both wild-type and scid/scid MEFs from the G_2_/M-phase fraction. The right side shows a box plot representation of the same data. Closed circles (•) indicate wild-type MEFs, and opened circles (○) indicate scid/scid MEFs. The numbers next to the circles indicate statistical significance compared to non-irradiated cells by *t-test*. The numbers in the box plot indicate statistical significance between wild-type MEFs and scid/scid MEFs by *t-test*. Each averaged I/A was calculated from 20 replicate experiments (4 independent experiments with 5 points taken in each experiment) for detecting relative ratios.

DNA-PKcs and the DNA-ligase IV/XRCC4 complex participate in the repair of DSBs in NHEJ, which is the main pathway for DNA repair [5]. The activity of DNA-PKcs was previously reported not to change throughout cell cycle, in cells irradiated by HDR [7]. Furthermore, increased number of γ*-*H2A.X foci due to defective DSB repair was observed in DNA-PKcs-deficient human cells at 24 h after high-dose γ*-*ray irradiation [19]. In the present study, by measuring the I/A ratio, we found cell cycle-dependent changes in the intensity of γ*-*H2A.X foci in wild-type and scid/scid MEFs at 1, 24, and 72 h after high-dose γ*-*ray irradiation (Fig. 4). More specifically, at 1 h after HDR γ*-*ray irradiation at a total dose of 4.32 Gy, the relative ratio (IR/non-IR) significantly increased both in wild-type and scid/scid G_1_- or G_2_/M-phase MEFs (Fig. 5A left, 5B left). Furthermore, the increased relative ratio (IR/non-IR) at 1 h after irradiation was significantly higher (*p*<0.001) in G_1_-phase scid/scid MEFs compared to wild-type MEFs (Fig. 5A, right). No significant difference was observed between the relative ratios of scid/scid and wild-type MEFs in the G_2_/M-phase (Fig. 5B, right). At 24 h after HDR γ*-*ray irradiation, the relative ratio of G_1_-phase scid/scid MEFs was significantly higher (*p*<0.01) than that of non-irradiated cells, although no significant difference was observed between irradiated and non-irradiated G_1_-phase wild-type MEFs (Fig. 5A left). Furthermore, the relative ratio in G_1_-phase scid/scid MEFs was significantly higher (*p*<0.05) than that in wild-type MEFs (Fig. 5A right). In contrast, the relative ratio in G_2_/M-phase scid/scid MEFs was not significantly different compared with wild-type MEFs (Fig. 5B, right). At 72 h after HDR γ*-*ray irradiation, relative ratios (IR/non-IR) from both wild-type and scid/scid MEFs were comparable in both the G_1_- and G_2_/M-phase (Fig. 5A, Fig 5B). These results indicate that γ*-*H2A.X foci intensity was significantly higher in G_1_-phase scid/scid MEFs compared to wild-type MEFs at 1 and 24 h after high-dose γ*-*ray irradiation, although such a difference is not observed in G_2_/M-phase MEFs. These results indicate that HDR-irradiation-induced DSBs can be repaired within 24 h in the mutation of DNA-PKcs in the G_2_/M-phase but not in the G_1_-phase MEFs.

### Time-course-dependent Increase in the Intensity of γ-H2A.X Foci in Response to MDR (0.06 Gy/h) γ-ray Irradiation

**Figure 6:**
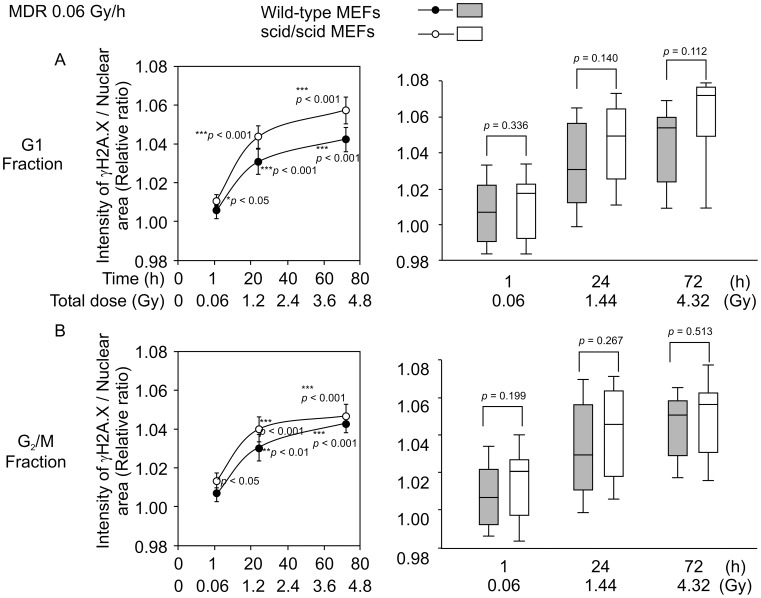
Time-course-dependent changes in the intensity of γ-H2A.X foci in response to MDR (0.06 Gy/h) γ-ray irradiation. A. The left side shows relative ratios (IR/non-IR) at 1, 24, and 72 h for MDR (0.06 Gy/h, and total doses were 0.06, 1.44, and 4.32 Gy, respectively) γ*-*ray irradiations in both wild-type and scid/scid MEFs from the G_1_-phase fraction. The right side is a box plot representation of the same data. B. The left side shows the relative ratios (IR/non-IR) at 1, 24, and 72 h in response to MDR (0.06 Gy/h, and total doses are 0.06, 1.44, and 4.32 Gy, respectively) γ*-*ray irradiations in both wild-type and scid/scid MEFs from the G_2_/M-phase fraction. B. The right side is a box plot representation of the same data. Closed circles (•) indicate wild-type MEFs, and open circles (○) indicate scid/scid MEFs. The numbers next to the circles indicate statistical significance compared to non-irradiated cells by *t-test*. The numbers in the box plot indicate statistical significance between wild-type MEFs and scid/scid MEFs by *t-test*. Each averaged I/A was calculated from 20 values (4 independent experiments with 5 points taken in each experiment) for detecting relative ratios.

We analyzed the changes induced in the fluorescence intensity of γ*-*H2A.X foci in MDR (0.06 Gy/h) γ*-*ray-irradiated wild-type and scid/scid MEFs at 1, 24, and 72 h after irradiation (Fig. 4). When cells were irradiated with MDR (0.06 Gy/h) irradiation for 1 h (total dose: 0.06 Gy), relative ratios (IR/non-IR) of scid/scid MEFs were significantly higher (*p*<0.05) than those of non-irradiated cells in the G_1_ and G_2_/M phase (Fig. 6A, left; 6B, left). In contrast, no significant difference was observed in wild-type MEFs (Fig. 6A, left; 6B, left). When cells were irradiated with MDR (0.06 Gy/h) irradiation for 24 and 72 h (total dose: 1.14 Gy and 4.32 Gy, respectively), the relative ratios in both wild-type and scid/scid MEFs in the G_1_ and G_2_/M phase were significantly higher (*p*<0.01) than those of non-irradiated MEFs (Fig. 6A, left; 6B, left). Furthermore, the relative ratios for scid/scid MEFs in response to the MDR γ*-*ray irradiations in both the G_1_ and G_2_/M phases were higher than those for wild-type MEFs, although no significant differences were detected (Fig. 6A, right; 6B, right). These results indicate that following MDR γ*-*ray irradiation at 0.06 Gy/h, the intensity of γ*-*H2A.X foci of both wild-type and scid/scid MEFs in the G_1_ or G_2_/M phase is increased in a total dose- and time-dependent manner (Fig. 6A, right; 6B, right). Our findings also illustrate that γ*-*H2A.X foci can form in MDR (0.06 Gy/h) γ*-*ray irradiated MEFs in both G_1_- and G_2_/M-phase cells, even in the mutation of DNA-PKcs. The effect of increased total radiation dose on the gradual phosphorylation of H2A.X is also independent of the activity of DNA-PKcs in both the G_1_ and G_2_/M phase.

### Time-course-dependent Increase in the Intensity of γ-H2A.X Foci in Response to MDR (0.015 Gy/h) γ-ray Irradiation

**Figure 7:**
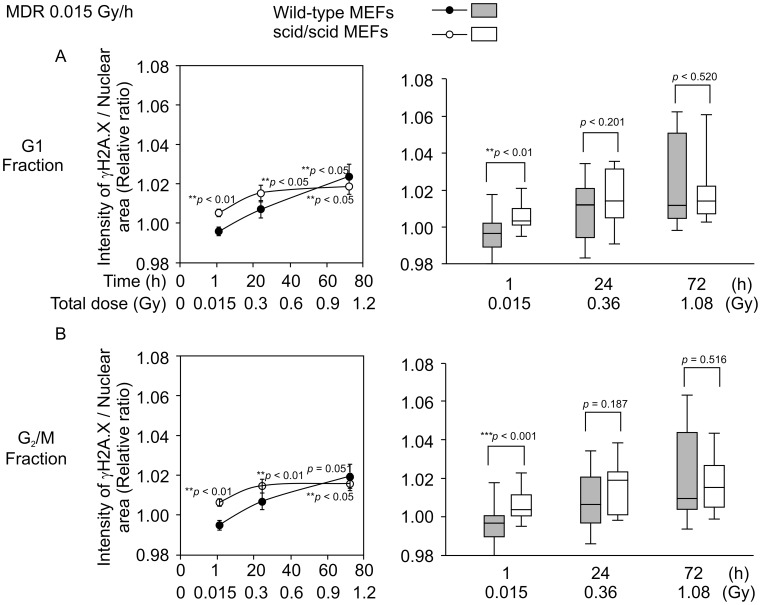
Time-course-dependent changes in the intensity of γ-H2A.X foci in response to MDR (0.015 Gy/h) γ-ray irradiation. A. The left side shows relative ratios (IR/non-IR) at 1, 24, and 72 h for MDR (0.015 Gy/h, and total doses are 0.015, 0.36 and 1.08 Gy, respectively) γ*-*ray irradiations in both wild-type and scid/scid MEFs from the G_1_-phase fraction. A. The right side is a box plot representation of the same values. B. The left side shows relative ratios (IR/non-IR) at 1, 24, and 72 h for MDR (0.015 Gy/h, and total doses are 0.015, 0.36, and 1.08 Gy, respectively) γ*-*ray irradiations in both wild-type and scid/scid MEFs from the G_2_/M-phase fraction. B. The right side is a box plot representation of the same data. Closed circles (•) indicate wild-type MEFs, and open circles (○) indicate scid/scid MEFs. The numbers next to the circles indicate statistical significance compared to non-irradiated cells by *t-test*. The numbers in the box plot indicate statistical significance between wild-type MEFs and scid/scid MEFs by *t-test*. Each averaged I/A was calculated from 20 obtained values (4 independent experiments with 5 points taken in each experiment) for detecting relative ratio.

We measured the fluorescent intensity of γ*-*H2A.X foci in MDR (0.015 Gy/h) γ*-*ray irradiated wild-type and scid/scid MEFs at 1, 24, and 72 h (Fig. 4). When cells were irradiated with MDR (0.015 Gy/h) γ-rays for 1 h (total dose: 0.015 Gy), relative ratios (IR/non-IR) from scid/scid MEFs were significantly higher (*p*<0.05) than those of non-irradiated cells in the G_1_ and G_2_/M phase (Fig. 7A, left; 7B, left), whereas those from wild-type MEFs showed no significant difference (Fig. 7A, left; 7B, left). Significant difference between scid/scid MEFs and wild-type MEFs at 1 h was observed in both cell cycle phases (Fig. 7A, right; 7B, right). In cells subject to MDR (0.015 Gy/h) irradiation for 24 h (total dose: 0.36 Gy), only scid/scid G_1_- and G_2_/M-phase MEF relative ratios (IR/non-IR) were significantly higher (*p*<0.05) than those of non-irradiated MEFs (Fig. 7A left, 7B left). In contrast, no significant difference was observed between scid/scid and wild-type MEFs in either cell cycle phase (Fig. 7A right, 7B right). When cells were subject to MDR (0.015 Gy/h) irradiation for 72 h (total dose: 1.08 Gy), relative ratios (IR/non-IR) of both wild-type and scid/scid MEFs in the G_1_ and G_2_/M phase were significantly higher (*p*<0.05, *p* = 0.051; only scid/scid/−MEFs in the G_2_/M phase) than those of non-irradiated cells (Fig. 7A left, 7B left). No difference was observed between wild-type and scid/scid MEFs in either cell cycle phase (Fig. 7A right, 7B right). These results indicate that the intensity of γ*-*H2A.X foci from both MEFs in both the G_1_ and G_2_/M phase subject to MDR (0.015 Gy/h) γ*-*ray irradiation is increased at a high total dose (1.08 Gy; Fig. 7A, left; 7B, left). Furthermore, these results suggest that DSBs induced by MDR (0.015 Gy/h) irradiation could be repaired by DNA-PKcs in wild-type MEFs, although they might remain unrepaired in scid/scid MEFs. Relative ratios of scid/scid and wild-type MEFs were comparable at 72 h following MDR (0.015 Gy/h) irradiation. This result might indicate that γ*-*H2A.X foci at later phases accumulated in a dose–dependent manner, and that accumulated foci might not be repaired via NHEJ as the mutation of DNA-PKcs has no effect on the increased foci intensity.

## Discussion

A large number of cells were used to detect differences in the fluorescence intensity of γ*-*H2A.X foci following LD or MDR γ*-*ray irradiation. Manual counting of γ*-*H2A.X foci in thousands of cells in experiments of LD or MDR γ*-*ray irradiation is tedious and leads to inconsistent results among researchers. The difficulty in manual counting of γ*-*H2A.X foci in LD or MDR γ*-*ray irradiation experiments is due to the inability to distinguish the weak intensity of γ*-*H2A.X foci from background levels. In this experiment, we show that counting using a cell function imager can be helpful when counting a large number of cells within a short period of time. Moreover, the cell function imager can evaluate the DNA content of each cell in cells fixed on tissue slides, and be used for analyzing cells fractionated in cell cycle phases by DAPI staining intensity. This device also allows the analysis of relations between cell cycle phase and γ*-*H2A.X foci intensity. In the experiments described herein, we measured the fluorescence intensity of whole γ*-*H2A.X foci in nuclei instead of counting the number of foci per cell, and we are proposing this as a new method to measure the intensity of γ*-*H2A.X foci in MDR irradiation studies. Therefore, the present method using the cell function imager can be a powerful tool for the detection of γ*-*H2A.X foci in LD or MDR γ*-*ray irradiation experiments. Previously, the analysis of γ-H2A.X based on fluorescent activated sorting (FACS) were reported using floating cells such as T-cells and lymphoblast cells lines [20], [21]. Analysis of γ-H2A.X foci in floating cells by FACS is more suitable than cell function imager. However, the analysis of γ-H2A.X foci by cell function imager analysis is superior to FACS analysis using solid state cells, because these are fractionated by cell nuclear image and do not require trypsinized treatment. Thus, our method of using cell function imager for γ-H2A.X foci in irradiated cells can be very useful.

Several published papers have addressed the formation of γ*-*H2A.X foci following HDR irradiation, in the absence of ATM or DNA-PKcs activity. The number of γ*-*H2A.X foci in ATM^−/−^MEFs was smaller than that in wild-type MEFs at 30 min after HDR (10 Gy: 5.5 Gy/min) γ*-*ray irradiation, but that in DNA-PKcs−/− MEFs was not [10]. This result indicates that ATM is responsible for phosphorylation of γ*-*H2A.X foci following HDR irradiation, but DNA-PKcs is not.

In addition, the formation of γ-H2A.X foci in ATM−/−MEFs was delayed at 1 h after HDR (2 Gy: 8.5 Gy/min) γ-ray irradiation compared to wild-type MEFs; however, this was not the case in DNA-PKcs−/−MEFs [11]. The formation of γ-H2A.X foci in ATM−/−MEFs was completely inhibited by DNA-PKcs inhibitor (LY294002) [11] indicating that both ATM and DNA-PKcs are dispensable for the formation of γ-H2A.X foci by HDR-irradiation. Furthermore, γ*-*H2A.X foci can be observed in all cell cycle phases at 24 h after 1 Gy of HDR irradiation in NHEJ-deficient cells, such as CHO-mutant V3 cells (DNA-PKcs-defective cells), which suggests that DNA repair is less effective at 24 h after HDR irradiation in NHEJ-deficient than in wild-type cells [8]. In the present study, the relative ratios of wild-type MEFs in the G_1_ phase were found to be significantly lower than those of scid/scid MEFs at 1 and 24 h after HDR irradiation (Fig. 5A). In contrast, the relative ratios of wild-type MEFs in the G_2_/M-phase were almost the same as those of scid/scid MEFs. These results indicate that DNA repair following HDR γ*-*ray irradiation is highly dependent on DNA-PKcs activity in G_1_-phase cells, as mutation of DNA-PKcs causes impaired NHEJ and delayed DNA repair. In addition to our preliminary experiments, ATM−/−MEFs could form γ-H2A.X foci by HDR-irradiation, and the relative ratios of wild-type MEFs in G_1_ phase were also found to be lower than ATM−/−MEFs at 1 and 24 h after HDR irradiation (Data not shown). Conversely, the relative ratios of wild-type MEFs in the G_2_/M-phase were almost the same as those of ATM−/−MEFs (Data not shown). These results also indicate that DNA repair following HDR γ-ray irradiation is dependent on ATM activity in G_1_-phase cells, as ATM deficiency causes impaired cell cycle check point and delayed DNA repair. Moreover, HR-deficient cells, such as CHO irs1SF cells, which have defective XRCC3, had no effect on DNA repair in the G_2_/M phase at 24 h after 1 Gy of HDR irradiation, because HR is important for DNA repair in the late S/G2 phase [8]. These results suggest that HR in the late S/G2 phase after HDR γ*-*ray irradiation efficiently repairs DNA damage even in the case of DNA-PKcs deficiency. Findings of our study indicate that intensity of γ*-*H2A.X foci in the G_2_/M-phase were not decreased by the mutation of DNA-PKcs or ATM deficiency after 24 h of HDR γ*-*ray irradiation. It is also suggested that DSBs might be efficiently repaired by HR in the G_2_/M-phase, which could compensate for defective NHEJ or ATM.

Previously, γ*-*H2A.X foci formation has been observed in MDR (0.018 Gy/h) irradiated human ATM-deficient cells in the G_0_/G_1_-phase [15]. We have also observed formation of γ*-*H2A.X foci in MDR (0.06 Gy/h) irradiated ATM^−/−^MEFs (data not shown). These results indicate that, in contrast to the case of HDR γ*-*ray irradiated cells, ATM is not always the major kinase for γ*-*H2A.X foci formation following MDR γ*-*ray irradiation. The ATM activated p53 in G_1_-phase causes the interference of entering S-phase [22], while the ATM and ATR activation in G_2_-phase causes the interference of entering M-phase to inhibit activation of CyclinB and Cdc2 complex [22]. Therefore, these results suggest that the G_1_ and G_2_ check points by ATM do not work appropriately for MDR-irradiated cells. Moreover, formation of γ*-*H2A.X foci could also be detected in MDR (0.06 and 0.015 Gy/h) irradiated scid/scid MEFs (Fig. 6A, left; 6B, left; 7A, left; 7B, left). Thus, γ*-*H2A.X foci formation or disappearance following MDR γ*-*ray irradiation might be caused by activation or de-phosphorylation of other PI3-kinases besides ATM or DNA-PKcs, such as ATR [23], [24].

Comparison of relative ratios (IR/non-IR) following MDR (0.06 Gy/h) γ*-*ray irradiation revealed higher values in G_1_-phase scid/scid MEFs than in wild-type MEFs (Fig. 6A, right), although the difference was not statistically significant, due to wide data deviation. These results indicate that in MDR (0.06 Gy/h) irradiated G_1_-phase cells, NHEJ may have a small impact on DNA repair.

Relative ratios (IR/non-IR) for γ*-*H2A.X foci after MDR (0.015 Gy/h) γ*-*ray irradiation in wild-type MEFs were significantly lower than those of scid/scid MEFs, at both the G_1_ and G_2_/M phases at 1 h of irradiation (total dose = 0.015 Gy; Fig. 7A, right; 7B, right). This result indicates that in MDR (0.015 Gy/h) irradiated wild-type MEFs, DNA-PKcs can repair DSBs in both the G_1_ and G_2_/M phase at 1 h. No difference in relative ratio (IR/non-IR) was observed between wild-type MEFs and scid/scid MEFs at 24 h and 72 h in the case of MDR at 0.015 Gy/h (Fig. 7A, right; 7B, right). A previous report has shown that dividing primary human fibroblast cells can repair DNA damage following LD γ*-*ray irradiation (0.0012 Gy by HDR irradiation), but non-dividing cells cannot [14]. In contrast, our results indicate that DSBs can be repaired in the G_1_ phase in non-dividing wild-type MEFs following MDR irradiation (0.015 Gy/h) at 1 h (0.015 Gy). These different results might be due to the difference in radiation dose-rate used in each study, namely, chronic MDR (0.015 Gy/h) as opposed to HDR irradiation for several seconds.

It is known that ATM phosphorylates p53 on ser15/18 following HDR irradiation, leading to cell cycle arrest at the G_1_ phase through activation of cdk inhibitors [25]. Additionally, HDR irradiation caused up-regulations of interferon stimulated genes in ATM deficient cells independently with p53 pathway [26]. Consistent with our previous report, activation of p53-dependent gene expression was observed in NIH/3T3Luc cells irradiated by γ*-*ray at MDR between 0.015 Gy/h and 0.09 Gy/h [27], [28], indicating that DNA repair is activated in MEFs even in these MDR irradiation doses. In our previous studies, p53 was not found to be phosphorylated on ser15/18 in MEFs after MDR irradiation [28]. Moreover, Ishizaki K et al. have reported that p53 is not phosphorylated on ser15 in human cells following MDR irradiation [29]. In our previous study, MDR-irradiated MEFs and whole spleen in mice demonstrated no phosphorylation of p53 on ser15/18, although enhanced p53-dependent gene expression was observed following continuous γ*-*ray irradiation at MDR (0.015–0.09 Gy/h) for 4 to 8 Gy [28], [30]. These observations indicate that p53 is activated by continuous γ*-*ray irradiation at MDR without phosphorylation on ser15/18 by ATM. Similarly, it is possible that phosphorylation of γ*-*H2A.X in mice might be enhanced by MDR (0.0164 Gy/h) γ*-*ray irradiation, also independent of ATM activity.

The intensity of γ*-*H2A.X foci in MDR-irradiated mice can be measured by the new method presented hereby, to evaluate L/MDR radiation risk. Measuring the intensity of γ*-*H2A.X foci by the cell function imager using this newly developed method will be applicable for assessment of the biological effects of LD or MDR γ*-*ray irradiation. This method allows for rapid and accurate analysis. Furthermore, our results clarified that formation of γ*-*H2A.X foci in response to continuous MDR γ*-*ray irradiation are increased by increment of total dose and irradiation time.

## Materials and Methods

### Establishment of MEFs and Cell Culture

Wild-type DNA-PKcs (FOX CHASE SCID C.B-17/lcr-+/+Jcl) or DNA-PKcs mutated (scid/scid) MEFs were derived from 12- to14–day-old mouse embryos in the C.B.17/DNA-PKcs^+/+^Jcl or FOX CHASE SCID C.B-17/lcr-^scid/scid^Jcl (CLEA Japan, Corporation, Japan) background, respectively. A responsible gene in the scid/sicd mouse was identified as a mutat of DNA-PKcs gene at Tyr-4046 [17]. Genotyping for scid/scid MEFs was performed using previously described method (Fig. S1) [17]. All experiments were conducted in accordance with the legal regulations in Japan and the guidelines for Animal experiments of the IES (Approval numbers: 21–14, 22–17 and 23–15, Approval date: 3/26/2009, 3/30/2010 and 3/28/2011).

### High- and Medium-dose-rate ^137^Cs-γ-ray Irradiation

One hundred microliters of each cell suspension (5–10×10^4^ cells/ml) of MEFs was seeded into each well of 96-well black plates (Griner, Frickenhausen, Germany), and cells were cultured in a CO_2_ incubator at 37°C for 3 days outside the irradiation room. The MEFs inside the CO_2_ incubator were irradiated with MDR ^137^Cs-γ*-*rays (Gamma-Simulator; Fuji Electronic Systems Company, Japan) at dose rates of 0.015 Gy/h and 0.06 Gy/h for 3 days, as previously described [19], [20]. Irradiation doses of ^137^Cs-γ*-*rays were measured using a photo-luminescent dosimeter (Asahi Technoglass Corporation, Japan). HDR irradiation with ^137^Cs-γ*-*ray was performed at a dose rate of 54 Gy/h for total doses of 4.32 Gy by Gamma-Cell 40 (MDS Nordion, Canada). For γ*-*H2A.X foci measurement, MEFs were fixed with 4% paraformaldehyde 1 h after the HDR γ*-*ray irradiation. Similarly, MEFs irradiated by chronic MDR γ*-*rays were fixed with 4% paraformaldehyde after 1, 24, and 72 h of MDR γ*-*ray irradiation. HDR-irradiated MEFs were also fixed at 1, 24, and 72 h after the HDR γ*-*ray irradiation for comparison with MDR-irradiated MEFs. According to the UNSCEAR report, low-dose-rate (LDR) and medium-dose-rate (MDR) were defined as ≤0.006 Gy/h and 0.006–6.00 Gy/h, respectively [31]. We used medium-dose rate (MDR) of 0.015–0.06 Gy/h according to the UNSCEAR definition.

### Immunocytochemical Analysis of γ-H2A.X Foci

Fixed cells were incubated with anti-γ*-*H2A.X antibody (Millipore Corp., Bedford, Mass., USA) for 1 h at 37°C, followed by incubation with Alexa647-conjugated anti-mouse IgG (Molecular Probe, USA) for 1 h at 37°C. Nuclei were stained with 4′,6-diamino-2-phenylindole (DAPI). The cell-function-imager (IN Cell Analyzer 1000; GE Healthcare BioScience, USA) automatically obtained simultaneous images of both DAPI and Alexa647 staining. A total of 45 images were obtained from 5 separate wells in a 96-well black plate, with 9 images per well obtained for each total dose and genotype. We used the Developer equipped IN Cell Analyzer 1000 (GE Healthcare BioScience, USA) computer software, which automatically recognizes the nuclear image stained by DAPI and measures intensity and surface area in each nucleus (Fig. 1B). Histograms of DAPI intensity and number of DAPI-stained cells were drawn using Excel XP (Microsoft, USA) (Fig. 2A, 3A), and detected cells were fractionated by DAPI intensity into the G_1_ or G_2_/M phase. The fractions of G_2_/M phase were confirmed by immune-staining using fluorescent intensity of anti-histone H3 (Ser10) (Millipore Corp., Bedford, Mass., USA). In the present study, H2A.X phosphorylation status in each cell was assessed by total fluorescent intensity of γ*-*H2A.X per nuclear area (I/A) instead of counting the number of γ*-*H2A.X foci in the nucleus. Average I/A was used as a representative value for each experimental condition. Furthermore, an average I/A from 20 replicates, derived from 5 wells in 4 independent experiments (5 well × 4 times), was confirmed by *t-test*. Statistical parametric analysis by *t-test* can be used in these experiments, because the average I/A did not follow an inclined distribution.

## Supporting Information

Figure S1 **The point mutation of DNA-PKcs in scid/scid MEFs.** The point mutation of DNA-PKcs in scid/scid MEFs was identified using restriction digestion method reported in the previous studies. After PCR amplification using the following primers: m6-DNA-PKcs(+), 5′-GGAAAAGAATTGGTATCCAC-3′; and m8-DNA-PKcs (-), 5′GTTGGCCCCTGCTAA CTTTC-3′, the DNA was digested using a restriction enzyme (*AluI*) [17]. The PCR fragments in scid/scid mice were digested at 38- and 26-bp, but not in C.B.17+/+ mice 64bp. Samples were resolved by electrophoresis with 2% NuSieve agarose (Cambrex Bio Science, USA). The numbers 1, 2 below scid/scid MEFs indicates a sample of individual mouse.
(TIF)
