# Supplementary figures and images for: Increased γ-H2A.X Intensity in Response to Chronic Medium-Dose-Rate γ-Ray Irradiation
Source: PLoS One. 2012 Sep 18;7(9):e45320. doi: 10.1371/journal.pone.0045320 (PMC3445451; doi:10.1371/journal.pone.0045320)

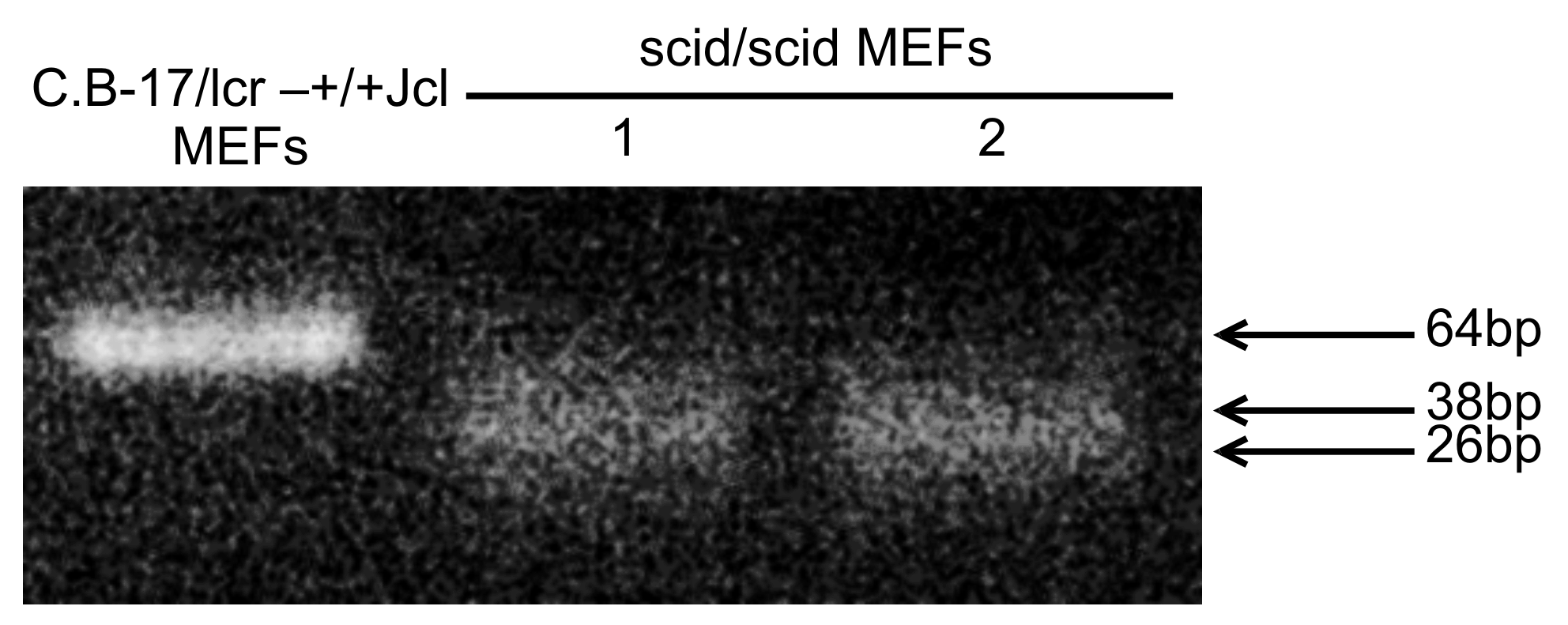

Supplement: Figure S1 — The point mutation of DNA-PKcs in scid/scid MEFs. The point mutation of DNA-PKcs in scid/scid MEFs was identified using restriction digestion method reported in the previous studies. After PCR amplification using the following primers: m6-DNA-PKcs(+), 5′-GGAAAAGAATTGGTATCCAC-3′; and m8-DNA-PKcs (-), 5′GTTGGCCCCTGCTAA CTTTC-3′, the DNA was digested using a restriction enzyme (AluI) [17]. The PCR fragments in scid/scid mice were digested at 38- and 26-bp, but not in C.B.17+/+ mice 64bp. Samples were resolved by electrophoresis with 2% NuSieve agarose (Cambrex Bio Science, USA). The numbers 1, 2 below scid/scid MEFs indicates a sample of individual mouse. (TIF) [file pone.0045320.s001.tif]
